# Higher levels of oxidative balance score linked to lower risk of gallstones: findings from the 2017–2020 National Health and Nutrition Examination Survey

**DOI:** 10.3389/fnut.2025.1521882

**Published:** 2025-01-29

**Authors:** Ting Xiong, Zhonghuo Chen, Jianwei Yi, Taozhi Yu, Kai Wang

**Affiliations:** ^1^Division of Hepato-Biliary-Pancreatic Surgery, Department of General Surgery, the Second Affiliated Hospital, Jiangxi Medical College, Nanchang University, Nanchang, China; ^2^Jiangxi Province Engineering Research Center of Hepatobiliary Disease, the Second Affiliated Hospital, Jiangxi Medical College, Nanchang University, Nanchang, China; ^3^Jiangxi Provincial Key Laboratory of Molecular Medicine, the Second Affiliated Hospital of Nanchang University, Nanchang, China; ^4^The MOE Basic Research and Innovation Center for the Targeted Therapeutics of Solid Tumors, the Second Affiliated Hospital, Jiangxi Medical College, Nanchang University, Nanchang, China; ^5^Jiangxi Provincial Clinical Research Center for General Surgery Disease, the Second Affiliated Hospital, Jiangxi Medical College, Nanchang University, Nanchang, China

**Keywords:** oxidative balance score, gallstone, NHANES, retrospective study, cross-sectional study

## Abstract

**Background and objective:**

The Oxidative Balance Score (OBS) has been linked to various chronic diseases; however, its association with gallstone prevalence remains underexplored. This study aimed to investigate the relationship between OBS and gallstone risk.

**Methods:**

This cross-sectional study utilized data from the National Health and Nutrition Examination Survey (NHANES) conducted from 2017 to March 2020. Weighted logistic regression models were applied to examine the association between OBS and the prevalence of gallstones, complemented by subgroup and sensitivity analyses. Restricted cubic spline (RCS) was used to investigate the nonlinear association between OBS and the prevalence of gallstones.

**Results:**

A total of 5,382 participants were included, among whom 592 reported a history of gallstones. After adjusting for confounding factors, a significant negative association was observed between OBS and gallstone prevalence (quartile 4 vs. quartile 1: odds ratio [OR] 0.63, 95% confidence interval [CI] 0.43–0.90, *p* = 0.019). The RCS analysis further supported a negative linear relationship between OBS and gallstone risk (nonlinear *p* = 0.149). The findings of the subgroup analyses exhibited considerable consistency.

**Conclusion:**

This study identified a significant negative linear association between OBS and gallstone risk, suggesting that higher OBS levels are associated with a reduced likelihood of gallstone formation.

## 1 Introduction

Gallstones are solid deposits that form in the gallbladder and are primarily classified into cholesterol, pigment, or mixed gallstones, with cholesterol gallstones being the most prevalent type (1). While many individuals with gallstones remain asymptomatic, over 20% experience symptoms, which vary depending on the location of the stones (2, 3). Gallstones are a common cause of biliary colic, whereas stones in the bile duct frequently present with right upper quadrant or epigastric pain, often due to acute ductal dilation secondary to obstruction. Pain relief may occur if the stones pass into the duodenum or re-enter the dilated bile duct. However, obstruction of the common bile duct may trigger secondary infections, potentially leading to acute obstructive suppurative cholangitis—a life-threatening condition. Without prompt medical intervention, this can result in severe acid–base and electrolyte imbalances, septic shock, multi-organ failure, and even death (4, 5). Gallstone prevalence varies significantly across populations and is influenced by factors such as age, gender, ethnicity, and lifestyle choices, including smoking, alcohol consumption, and physical activity levels (6–9). Globally, studies estimate that gallstones affect approximately 11.2% of the adult population in South America, whereas prevalence rates as low as 5.1% have been reported in parts of Asia (10). These differences highlight the influence of demographics and lifestyle on gallstone risk (11). Furthermore, the economic burden of gallstone disease is substantial, encompassing costs associated with diagnosis, treatment, and surgical interventions, which collectively amount to billions of dollars annually in healthcare expenses (12).

Gallstone formation is a multifactorial process influenced by bile composition imbalances, gallbladder motility disorders, and dietary factors. Oxidative stress plays a pivotal role in various physiological processes, such as signal transduction and immune response, under normal conditions (13, 14). However, in pathological states, excessive production of reactive oxygen species (ROS) can overwhelm the body’s antioxidant defenses, leading to inflammation and cellular damage, which are key contributors to gallstone development (15–17). As such, understanding oxidative balance is crucial for elucidating the etiology of gallstones and developing early prevention strategies.

Oxidative balance refers to the equilibrium between pro-oxidants and antioxidants in the body, which influences numerous biological processes. Endogenous antioxidants, such as glutathione and superoxide dismutase, together with exogenous antioxidants like vitamins C and E, as well as β-carotene, play essential roles in mitigating oxidative stress (18). Conversely, pro-oxidants, including free radicals, can amplify cellular damage when their levels surpass the neutralizing capacity of antioxidants. The Oxidative Balance Score (OBS), a composite measure that integrates 16 dietary and 4 lifestyle factors, represents a valuable tool for assessing oxidative balance. By accounting for various components that affect oxidative stress and antioxidant levels, OBS provides a holistic and dynamic evaluation compared to individual antioxidant biomarkers (19). This multidimensional approach considers both the relative contributions of dietary and biochemical antioxidants and the influence of daily lifestyle habits on oxidative status, offering a more comprehensive reflection of an individual’s antioxidant capacity. Notably, a higher OBS signifies greater systemic antioxidant capacity. Emerging evidence suggests that higher OBS values may exert a protective effect against the development of chronic diseases (20–23), underscoring the potential of OBS as a predictive marker for health outcomes.

Oxidative stress has been suggested to play a role in gallstone formation (24, 25). However, the specific relationship between the OBS and gallstone risk remains poorly understood. Elucidating this association is crucial for identifying potential preventive strategies and therapeutic targets. Therefore, the objective of this study was to evaluate the association between OBS and gallstone prevalence using data from the National Health and Nutrition Examination Survey (NHANES) conducted from 2017 to March 2020. Through a comprehensive analysis, this study aimed to provide valuable insights into gallstone prevention by highlighting the potential protective effects of dietary antioxidants and healthy lifestyle habits.

## 2 Materials and methods

### 2.1 Study design and participants

**Figure 1:**
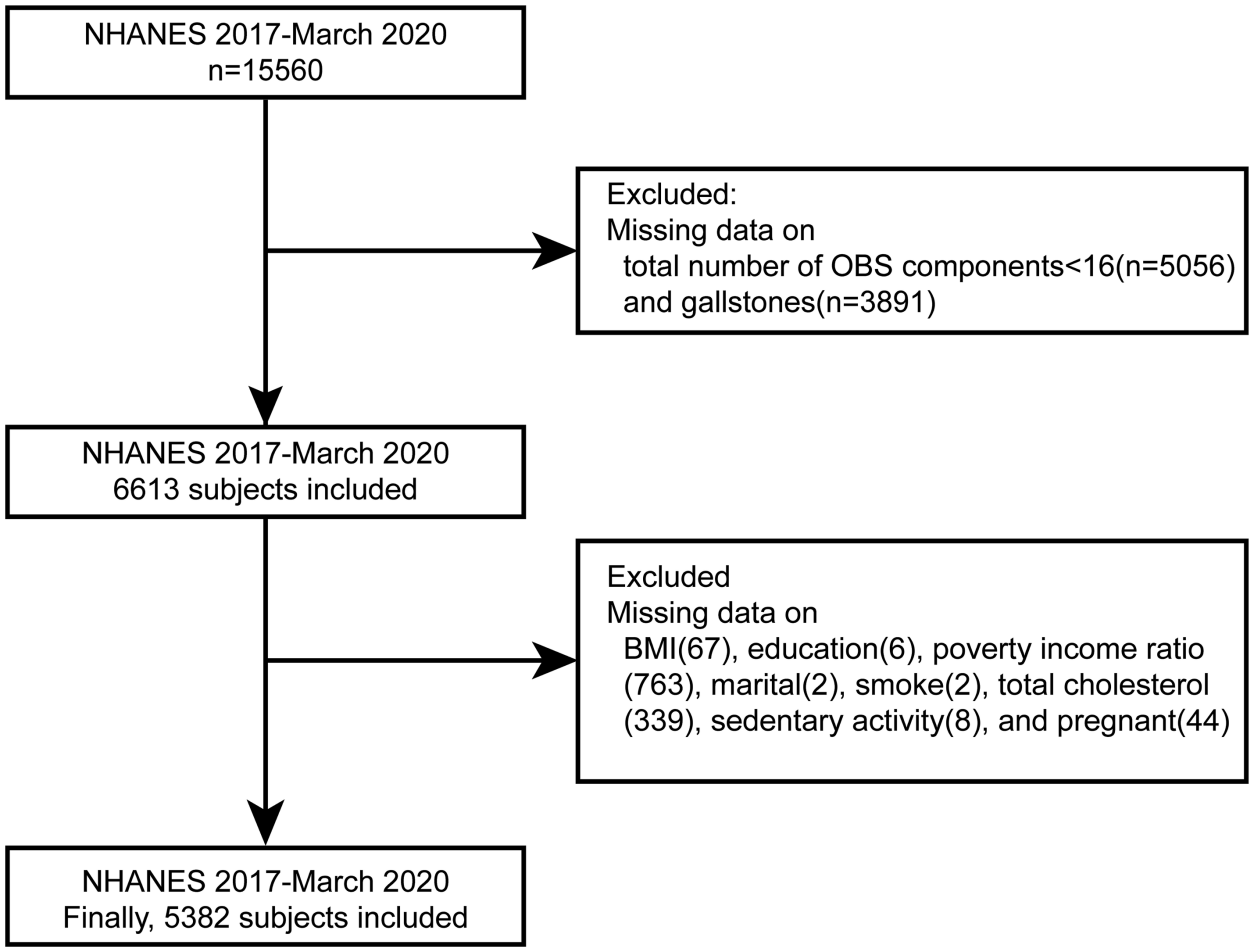
Flow chart of the study participants.

NHANES is a stratified, multistage sampling survey designed to assess the health and nutritional status of individuals across the United States. It aims to evaluate health and nutritional profiles in both adults and children, employing a stratified, multistage probability sampling design to recruit a representative sample of the civilian, noninstitutionalized U.S. population. The sampling framework segments the nation into various strata based on demographic and geographic characteristics. Within each stratum, clusters are identified, and a random sample of households is selected. The survey protocol was approved by the National Center for Health Statistics Institutional Review Board (NCHS IRB), and all participants provided informed written consent prior to participation. For this study, data were obtained from NHANES conducted between 2017 and March 2020, encompassing a total of 15,560 participants. Participants with missing data in key variables were excluded during screening, including individuals with more than four missing values in the OBS assessment components and those lacking data on gallstone status. After excluding participants with incomplete covariate data, a final sample of 5,382 individuals was included in the analysis (refer to Figure 1).

### 2.2 Data collection

We obtained data from the NHANES website for the period from March 2017 to March 2020. This dataset encompasses various information about the study participants, including age, gender, race, marital status, education level, poverty-income ratio, smoking status, and serum total cholesterol levels. Furthermore, we collected essential data necessary for the assessment of diabetes and hypertension, including blood pressure, blood glucose, glycated hemoglobin levels, and other relevant indicators. In terms of dietary and lifestyle factors, we gathered multiple data points, including dietary intake levels of fiber, β-carotene, vitamins B6, B12, C, and E, riboflavin, folate, niacin, calcium, magnesium, zinc, copper, and selenium. Additionally, we assessed iron intake, total fat intake, cotinine levels, and alcohol consumption. Physical activity levels and body mass index (BMI) were also incorporated into the evaluation. Together, these dietary and lifestyle data points constitute a comprehensive dataset for the assessment of health outcomes.

### 2.3 Assessment of OBS

Following established research protocols, the OBS was calculated using 16 nutrient components and 4 lifestyle factors, comprising 5 pro-oxidative and 15 antioxidative components (26). Antioxidative components included dietary intake levels of fiber, β-carotene, vitamins B6, B12, C, and E, riboflavin, folate, niacin, calcium, magnesium, zinc, copper, and selenium, as well as physical activity levels. In contrast, pro-oxidative components consisted of iron intake, total fat intake, smoking status, alcohol consumption, and obesity (BMI). Dietary intake data were averaged across two recorded days. Smoking status was evaluated via cotinine levels, while physical activity levels were assessed using methods established in prior studies (27). As for alcohol consumption, based on previous research, participants were categorized into non-drinkers, light drinkers, and heavy drinkers, receiving scores of 2, 1, and 0, respectively (26). All other factors, excluding alcohol intake, were stratified into tertiles by gender. For antioxidative components, participants in the highest tertile received 0 points, the middle tertile received 1 point, and the lowest tertile received 2 points. For pro-oxidative components, scoring was reversed, with participants in the highest tertile receiving 2 points. The OBS was computed as the sum of scores for all components, with a higher OBS indicating greater overall exposure to antioxidants. Additional details regarding the scoring algorithm are presented in Supplementary Table S1.

### 2.4 Gallstone assessment

The presence of gallstones was determined using responses from the Medical Conditions questionnaire. Participants were asked the question: “Has a doctor or other health professional ever informed you that you had gallstones?” Those who answered “yes” were classified as having gallstones, whereas participants who responded “no” were categorized as gallstone-free.

### 2.5 Covariates

This study accounted for various covariates, including age, race, marital status, education level, PIR, sugar intake, caffeine intake, water intake, diabetes, and hypertension. Additional details regarding the assessment of diabetes and hypertension are provided in Supplementary Table S2.

### 2.6 Statistical analysis

We conducted a detailed summary of baseline variables. For continuous data that were not normally distributed, we described these variables using medians and interquartile ranges (IQRs), while categorical variables were reported as counts and weighted percentages. To assess the relationship between the OBS and the presence of gallstones, we analyzed OBS in two forms: as a continuous variable and as a quartile-categorized variable. Differences in continuous variables across different OBS quartiles were evaluated using the Wilcoxon rank-sum test, whereas differences in categorical variables were assessed using the chi-square test.

Additionally, weighted logistic regression analyses were performed to explore the association between OBS, in both its continuous and quartile forms, and gallstone occurrence. Interaction analyses were conducted to investigate potential subgroup interactions, and Restricted cubic spline (RCS) curves were employed to examine the nonlinear associations between OBS and gallstones risk. The OBS was further categorized into dietary and lifestyle components, with each component analyzed using weighted logistic regression, complemented by RCS curves for visual representation. To ensure the robustness of our results, sensitivity analyses were conducted by sequentially removing each OBS component from the model. All statistical analyses were two-sided, with a significance level set at *p* < 0.05. All analyses were performed using R version 4.3.1, with “WTDR2DPP” used as a weighting variable.

## 3 Results

### 3.1 Baseline characteristics

In this study, a total of 5,382 adults were analyzed, of whom 592 were identified as gallstone patients (Table 1). Significant differences in most baseline characteristics were observed between the gallstone and non-gallstone groups. However, no significant differences were found in key parameters such as total cholesterol intake (*p* = 0.369), total sugar intake (*p* = 0.149), total caffeine intake (*p* = 0.777), and education level (*p* = 0.589). Demographic analysis revealed that gallstone patients were predominantly women (73.50% vs. 26.50%) and tended to be older than those without gallstones. Additionally, individuals with gallstones experienced a higher prevalence of unfavorable marital status (24.54% vs. 17.13%) and had lower income levels. Ethnic analysis showed that non-Hispanic White individuals were significantly more likely to have gallstones (*p* = 0.032). Furthermore, gallstones were notably more prevalent among individuals with a higher BMI (32.9 vs. 29.42). Lifestyle factors also differed significantly between the two groups. Gallstone patients had lower total water intake than non-gallstone individuals (*p* = 0.036) and exhibited longer periods of sedentary behavior (*p* = 0.020), higher rates of smoking exposure, and an increased prevalence of diabetes and hypertension (*p* < 0.001 for all). Importantly, patients with gallstones exhibited substantially lower OBS compared to those without gallstones (*p* = 0.013). Additional details on baseline characteristics stratified by OBS quartiles are provided in Supplementary Table S3.

**Table 1:** Baseline characteristics based on the presence or absence of gallstones.

Characteristic	Overall *n* = 5,382	Non gallstones *n* = 4,790	Gallstones *n* = 592	*p*-value
**Sex**				
Male	2,576 (48.41%)	2,411 (51.19%)	165 (26.50%)	<0.001
Female	2,806 (51.59%)	2,379 (48.81%)	427 (73.50%)	<0.001
Age	48.58 (17.23)	47.44 (17.09)	57.56 (15.62)	<0.001
**Marital**				
Married/living with partner	3,174 (63.30%)	2,815 (63.33%)	359 (63.04%)	0.005
Widowed/divorced/separated	1,213 (18.74%)	1,051 (17.13%)	162 (24.54%)	
Never married	995 (17.96%)	924 (19.54%)	71 (12.42%)	
**Race**				
Mexican American	606 (8.05%)	535 (8.18%)	71 (7.05%)	0.032
Non-Hispanic White	2,063 (64.71%)	1,781 (63.90%)	282 (71.15%)	
Non-Hispanic Black	1,394 (10.45%)	1,287 (10.91%)	107 (6.84%)	
Other	1,319 (16.79%)	1,187 (17.01%)	132 (14.96%)	
PIR	3.17 (1.64)	3.20 (1.65)	2.94 (1.60)	0.004
BMI	29.81 (7.08)	29.42 (6.76)	32.90 (8.62)	<0.001
Total cholesterol, mg/dL	188.13 (40.71)	187.84 (40.54)	190.41 (41.98)	0.369
Total sugars, gm	101.34 (62.92)	100.87 (62.54)	105.07 (65.73)	0.149
Total caffe, gm	169.52 (184.92)	169.53 (187.48)	169.43 (163.40)	0.777
Total water, gm	2928.77 (1267.93)	2947.38 (1272.65)	2781.72 (1221.11)	0.036
Sedentary activity, min	374.93 (607.80)	368.13 (576.32)	429.96 (817.76)	0.020
**Education**				
Under high school	830 (9.08%)	740 (9.22%)	90 (7.92%)	0.589
High school or equivalent	1,255 (26.83%)	1,107 (25.95%)	148 (33.76%)	
Above high school	3,297 (64.09%)	2,943 (64.82%)	354 (58.32%)	
**Smoke**				
Current smoke	940 (15.65%)	845 (15.62%)	95 (15.93%)	<0.001
Former smoke	1,352 (26.14%)	1,158 (25.26%)	194 (33.15%)	
Non smoke	3,090 (58.20%)	2,787 (59.12%)	303 (50.92%)	
**Diabetes**				
No	4,248 (84.27%)	3,857 (85.69%)	391 (73.04%)	<0.001
Yes	1,134 (15.73%)	933 (14.31%)	201 (26.95%)	
**Hypertension**				
No	2,911 (60.73%)	2,675 (63.20%)	236 (41.26%)	<0.001
Yes	2,471 (39.27%)	2,115 (36.80%)	356 (58.74%)	
OBS continuous	21.87 (6.93)	22.02 (6.88)	20.76 (7.21)	0.013

All values are presented as mean ± SD or as counts (weighted, proportion). OBS, oxidative balance score; PIR, poverty income ratio; BMI, body mass index.

### 3.2 Associations between OBS and gallstones

**Figure 2:**
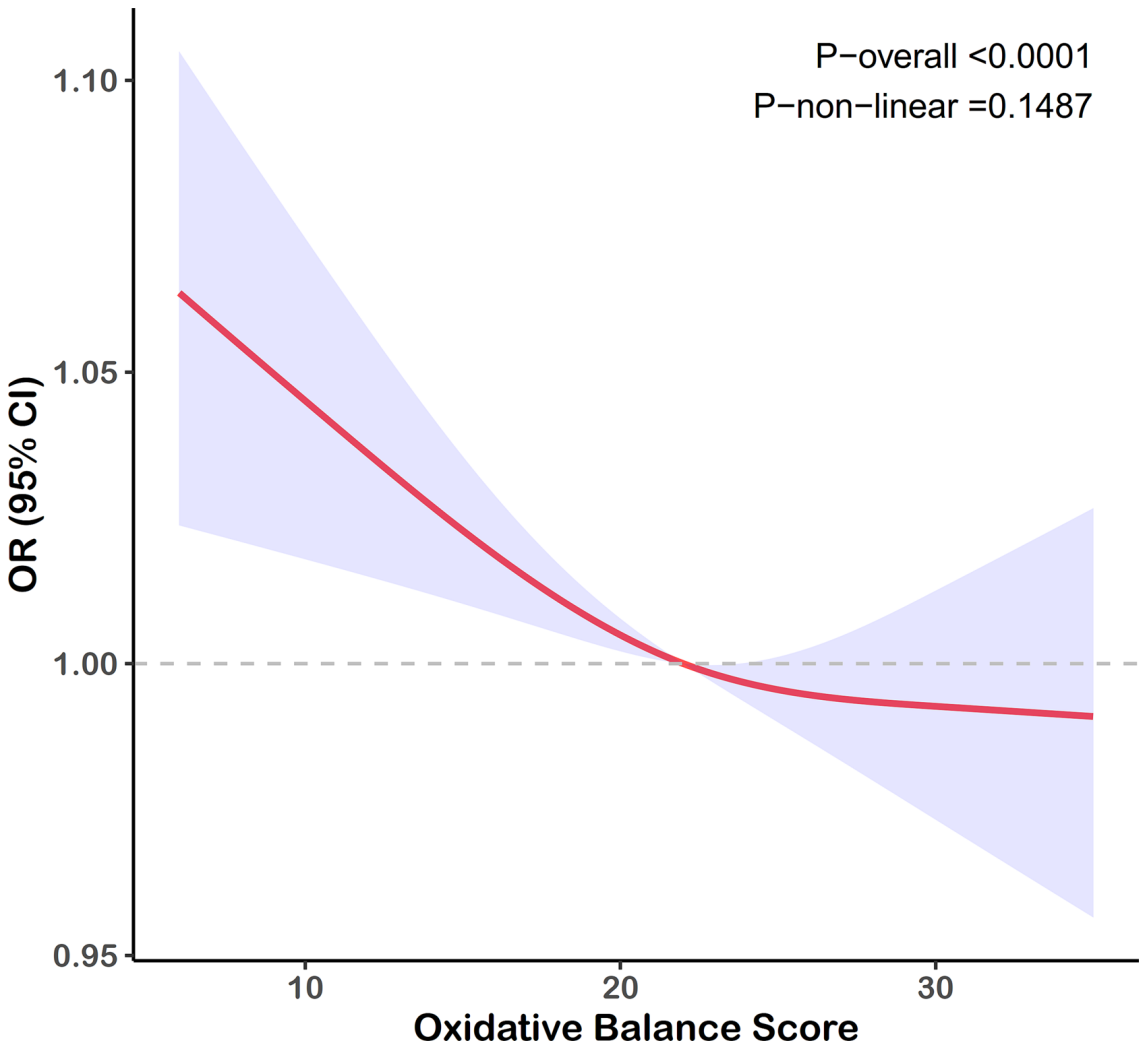
RCS curve for the association between the OBS and the risk of gallstones. Lines represent odds ratios, and areas represent 95% confidence intervals. The model was adjusted for age, race, marital, poverty income ratio, education, total sugars, total caffe, total water, diabetes, and hypertension.

A negative correlation was consistently observed between the OBS and gallstone risk across all models. As shown in Table 2, after adjusting for covariates, participants in the highest OBS quartile experienced an approximately 40% reduction in gallstone risk compared to those in the lowest quartile (95% confidence interval [CI]: 0.43–0.90), with similar trends observed in Models 1 and 2. When analyzed as a continuous variable, the OBS did not demonstrate a significant association with gallstone risk in Model 3. Figure 2 depicted a negative linear relationship between the OBS and gallstone prevalence, with a nonlinear *p*-value of 0.149.

**Table 2:** ORs (95% CIs) for gallstones according to the OBS.

Characteristic	Model 1			Model 2			Model 3		
	OR	95% CI	*p*-value	OR	95% CI	*p*-value	OR	95% CI	*p*-value
Continuous	0.97	0.96–0.99	0.010	0.97	0.96–0.99	0.005	0.98	0.96–1.00	0.066
**OBS quantile**									
Q1	–	–	–	–	–		–	–	
Q2	0.81	0.60–1.10	0.200	0.76	0.56–1.04	0.100	0.77	0.58–1.04	0.103
Q3	0.79	0.57–1.08	0.150	0.71	0.53–0.97	0.043	0.72	0.58–1.01	0.073
Q4	0.63	0.45–0.90	0.016	0.61	0.44–0.84	0.006	0.63	0.43–0.90	0.019
P for trend			0.024			0.013			0.050

OR, odds ratio; CI, confidence interval. Model 1: Unadjusted. Model 2: Adjusted for age, and race. Model 3: Adjusted for age, race, marital, poverty income ratio, education, total sugars, total caffe, total water, diabetes, hypertension.

### 3.3 Associations between dietary/lifestyle OBS and gallstones

We analyzed the association between overall OBS and gallstone risk by separating OBS into dietary and lifestyle components, as shown in Supplementary Table S4. A significant inverse association was observed between dietary OBS and gallstone prevalence in models 1 and 2. However, in Model 3, after comprehensive adjustments, this association weakened; while individuals in the third dietary OBS quartile (Q3) had a significantly lower risk of gallstones compared to those in the lowest quartile ([OR]: 0.58, 95% CI: 0.38–0.89, *p* = 0.019), the association between dietary OBS as a continuous variable and gallstone risk became non-significant ([OR]: 0.97, 95% CI: 0.95–1.00, *p* = 0.094). Similarly, lifestyle OBS showed a significant inverse association with gallstone prevalence in all models when analyzed as a continuous variable ([OR]: 0.88, 95% CI: 0.79–0.99, *p* = 0.042 in Model 3). Furthermore, individuals in the highest lifestyle OBS quartile (Q4) exhibited a significantly reduced risk of gallstones in Model 3 ([OR]: 0.60, 95% CI: 0.37–0.96, *p* = 0.046). Supplementary Figures S1, S2 demonstrate a consistent, negative trend between both dietary and lifestyle OBS components and gallstone prevalence, with nonlinear *p*-values of 0.151 and 0.680, respectively.

### 3.4 Subgroup analysis and sensitivity analyses

**Figure 3:**
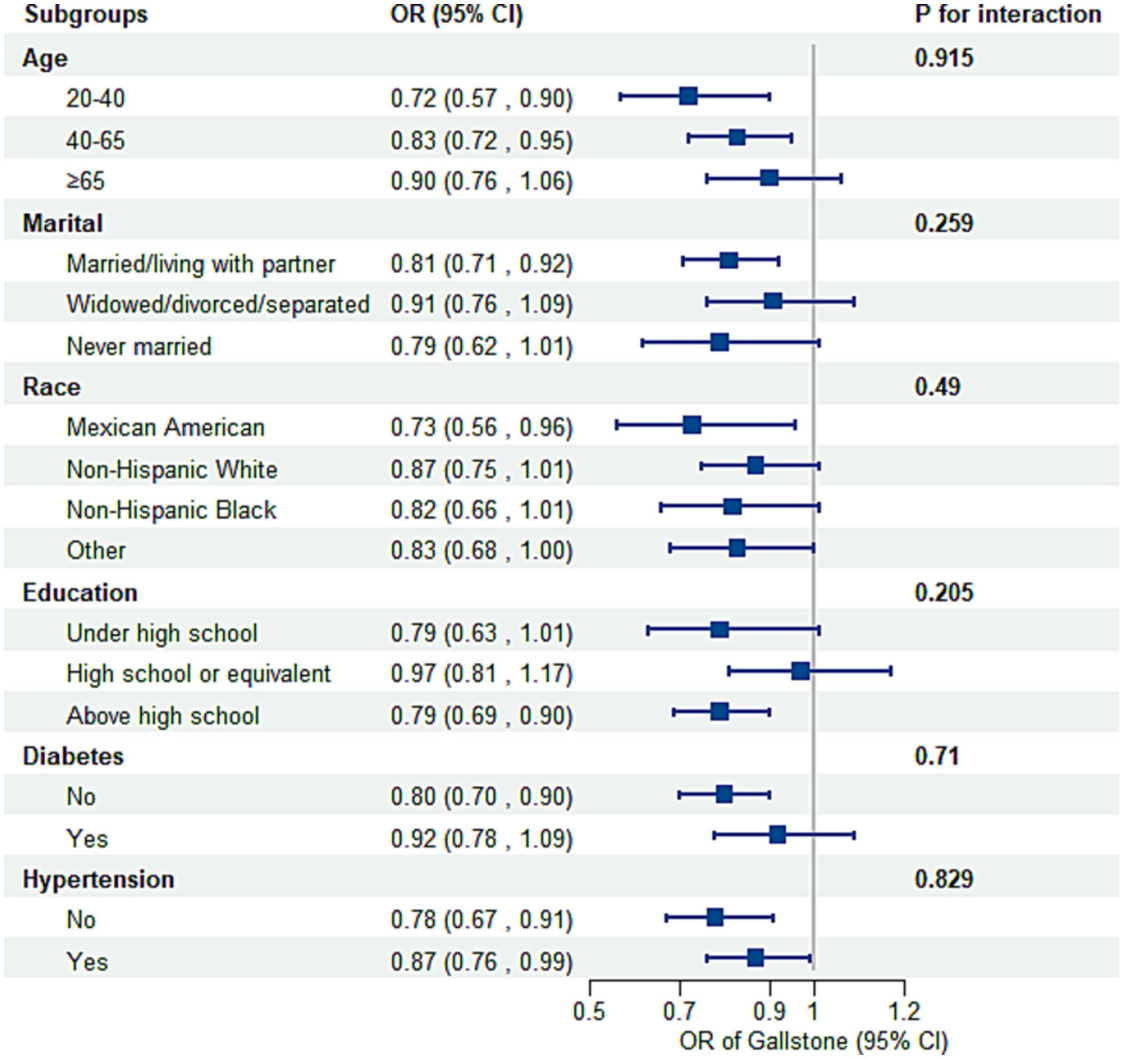
Subgroup analysis of the association between the OBS and the risk of gallstones. Adjusted for age, race, marital, poverty income ratio, education, total sugars, total caffe, total water, diabetes, and hypertension. OR, odds ratio; CI, confidence interval.

Subgroup analyses and interaction tests, stratified by age, marital status, race, education, diabetes, and hypertension status, were conducted to assess variations in the association between OBS and gallstone risk (Figure 3). No statistically significant interactions were found (P for interaction > 0.05). However, stratified analyses showed some variations in effect size. The inverse association was strongest among younger participants (20–40 years: OR = 0.72, 95% CI: 0.57–0.90; 40–65 years: OR = 0.83, 95% CI: 0.72–0.95) but weakened in those aged ≥65 years (OR = 0.90, 95% CI: 0.76–1.06, P for interaction = 0.915). Married or partnered individuals exhibited a significant inverse association (OR = 0.81, 95% CI: 0.71–0.92), while no significant association was observed in other marital groups. Mexican Americans showed the strongest negative association by race (OR = 0.73, 95% CI: 0.56–0.96), followed by other groups with weaker or non-significant associations. Participants with education above high school demonstrated a significant association (OR = 0.79, 95% CI: 0.69–0.90), whereas the association was weaker in those with lower education levels. Diabetes did not significantly modify the association (P for interaction = 0.710), though the association was stronger in individuals without diabetes (OR = 0.80, 95% CI: 0.70–0.90) compared to those with diabetes (OR = 0.92, 95% CI: 0.78–1.09). Despite minor variations, the overall inverse association between OBS and gallstone risk remained consistent across most subgroups. Sensitivity analyses further confirmed the robustness of these findings, with stable associations across various OBS components (Supplementary Table S5).

## 4 Discussion

This study analyzed a cohort of 5,382 adults from the NHANES database (2017 to March 2020) to evaluate the association between the OBS and gallstone prevalence. Logistic regression and trend analyses identified a significant inverse association between OBS and gallstone prevalence, with higher OBS values linked to a reduced risk of gallstones. Findings from dose–response analyses indicated a significant linear negative correlation between OBS and gallstone prevalence. When OBS was divided into dietary and lifestyle components, the results demonstrated an intriguing imbalance: lifestyle OBS exhibited a stronger and more consistent inverse association with gallstone prevalence, whereas dietary OBS showed a weaker and attenuated association, particularly in higher quantiles. Overall, our results indicated that enhancing OBS through dietary intake and lifestyle modifications may contributed to a reduced prevalence of gallstones.

The pathogenesis of gallstones is a complex and multifactorial process involving various factors such as environment, diet, and metabolism (28–30). Mechanistically, the formation of gallstones is typically related to the imbalance of bile components, specifically the dysregulation of cholesterol, bile salts, and phospholipid ratios within bile. This imbalance may lead to cholesterol supersaturation, resulting in the formation of solid crystals in the gallbladder. Additionally, gallbladder dysmotility, such as reduced gallbladder emptying capacity, can lead to bile stasis, creating favorable conditions for stone formation. Oxidative stress and inflammation are significant promoting factors in the formation of gallstones. Studies have shown that in gallstone patients, the gallbladder mucosa often exhibits elevated levels of oxidative stress, which can induce the oxidation of bilirubin and the polymerization of free radicals through the action of ROS, ultimately leading to the deposition of insoluble macromolecular polymers that promote stone formation (31, 32). Furthermore, oxidative stress can exacerbate the inflammatory response in the gallbladder. During the inflammatory process, the activation of immune cells such as neutrophils not only further triggers the accumulation of cholesterol and calcium crystals but may also damage gallbladder endothelial cells, affecting normal gallbladder function, such as reduced bile emptying capacity, thus exacerbating bile stasis and forming a vicious cycle (24, 25). Our research results indicate that high OBS has a protective effect on the development of gallstones, which is consistent with current understanding of the role of oxidative stress in the pathogenesis of gallstones.

Simultaneously, environmental factors and dietary habits, such as high-fat diets, low fiber intake, and adverse lifestyle choices (e.g., smoking and lack of exercise), indirectly influence the risk of gallstone formation by altering bile metabolism and oxidative stress levels in the body (8). A large-scale prospective cohort study indicates that adherence to the Alternative Mediterranean Diet score (aMED) and the 2015 Healthy Eating Index (HEI-2015) dietary patterns can reduce the risk of gallstone disease by 10% (33). Regarding dietary factors, some studies indicate that dietary fiber (34, 35), carotenoids (36), B vitamins (37), vitamin C (38, 39), vitamin E (40), magnesium (41), zinc (42), and copper (43) may help reduce the formation of gallstones. In contrast, the intake of pro-oxidants represented by fats is associated with the occurrence of gallstones (44). The role of lifestyle factors in the incidence of gallstones has been extensively studied. Evidence suggests that obesity and behaviors associated with smoking and alcohol consumption significantly elevate the risk of gallstone formation (45, 46). In obese individuals, adipocytes play a critical role by promoting cholesterol crystallization and gallstone formation through the secretion of leptin (47). Leptin exerts multifaceted effects on gallbladder physiology, including alterations in bile volume, sodium and pH regulation, as well as modulation of inflammatory cytokine-related and ion transport-related genes (48). Furthermore, obesity is frequently associated with insulin resistance, which dysregulates hepatic metabolism. Specifically, hepatic insulin resistance leads to the activation of the forkhead transcription factor (FoxO1), which promotes the overexpression of bile cholesterol transport proteins Abcg5 and Abcg8, resulting in excessive cholesterol secretion into bile. Simultaneously, it suppresses bile acid synthase (e.g., Cyp7b1) expression and interferes with the farnesoid X receptor (FXR) pathway, thus altering the bile salt composition in a manner that favors gallstone formation (49). Obesity also induces systemic inflammation similar to the effects of smoking and alcohol consumption, triggering heightened oxidative stress in the gallbladder, which damages endothelial cells and impairs gallbladder motility (50, 51). This hypocontraction further exacerbates bile stasis, facilitating gallstone development. Physical activity, on the other hand, has been shown to mitigate oxidative stress by reducing free radical levels, thereby preventing the onset of various chronic diseases (52). However, compared to lifestyle factors, dietary antioxidants alone appear to have a relatively weaker impact on mitigating oxidative damage. Studies indicate that a high level of dietary antioxidant intake does not consistently lead to a pronounced reduction in oxidative stress, even act as pro-oxidants (53). Our results corroborate this finding: the association between dietary OBS and gallstone risk was only significant at the Q2 level after controlling for relevant covariates. In contrast, lifestyle OBS demonstrated a more robust and significant negative association with gallstone risk. Interestingly, both dietary OBS and lifestyle OBS exhibited their strongest negative associations with gallstones at the Q2 level. However, RCS analysis did not reveal a non-linear relationship. Importantly, a combined OBS, integrating both dietary and lifestyle factors, demonstrated the strongest and most consistent protective association with gallstones. Thus, we propose that an integrated OBS combining dietary antioxidants and healthy lifestyle behaviors may offer superior preventive and monitoring capabilities for gallstones.

Subgroup analysis revealed heterogeneity in the association between OBS and gallstones across different subgroups. Notably, the association between OBS and gallstones became non-significant in populations aged 65 years or older. This could be attributed to the fact that older adults often experience heightened baseline oxidative stress. Aging is inherently accompanied by increased ROS production due to mitochondrial dysfunction, cellular senescence, and a decline in antioxidant defense mechanisms (54). Furthermore, multiple metabolic comorbidities common in older adults contribute additional sources of oxidative stress, which may overshadow the protective effects of OBS. Consequently, the oxidative stress background in elderly individuals might exceed the protective threshold of OBS, diminishing observable associations with gallstone risk. Additionally, gallstone formation in the elderly may be more influenced by non-oxidative stress-related mechanisms. For instance, slowed bile flow due to declining gallbladder motility and increased cholesterol saturation are significant age-related risk factors for gallstones (55). Moreover, although older adults may report higher antioxidant intake (e.g., through dietary supplements), issues related to absorption efficiency and metabolic limitations may reduce the actual antioxidant efficacy, thereby contributing to the non-significant association observed in this group (56). No significant association between OBS and gallstones was observed in populations with unfavorable marital statuses, such as widowed, divorced, or single individuals. This may be partially explained by the role of psychological and behavioral factors in mediating oxidative stress. Married or cohabitating individuals often demonstrate more stable lifestyle habits, such as healthier diets, more regular routines, and greater social support, all of which are associated with lower oxidative stress and potentially higher OBS scores. Conversely, single individuals or those in unhealthy marriages are more likely to experience heightened psychosocial stress, which has been shown to strongly correlate with elevated oxidative stress levels (57). This increased psychosocial stress may counteract the protective effects of a theoretically higher OBS. Additionally, these individuals often engage in less favorable health behaviors, such as poor dietary habits, inadequate physical activity, smoking, and alcohol consumption, which further exacerbate cholesterol metabolism disorders or bile stasis, masking the protective role of OBS. For instance, evidence indicates that smoking and high-fat diets significantly elevate gallstone risk, and these behaviors tend to be more prevalent in single or widowed individuals. The subgroup analysis of education levels similarly revealed discrepancies. The negative association between OBS and gallstones was significant only in populations with higher education levels. This could reflect disparities in health resource accessibility and health literacy. Individuals with lower educational attainment may have limited access to health-promoting resources and knowledge, leading to difficulties in adopting and sustaining health behaviors aligned with higher OBS scores (e.g., balanced diets and regular exercise). Furthermore, individuals with lower education levels are often subjected to greater life stressors, including economic disadvantages, which are associated with elevated oxidative stress. These factors collectively attenuate the potential protective effect of OBS in this group. In diabetic populations, no significant association between OBS and gallstones was observed. This may be explained by the inherently high baseline oxidative stress levels associated with diabetes, driven by chronic hyperglycemia, insulin resistance, and elevated ROS production (58). Such oxidative damage may diminish or even negate the protective effect of OBS. Furthermore, diabetes is known to disrupt bile secretion and metabolism, such as through impaired insulin-mediated bile signaling, leading to bile composition changes that increase gallstone risk (59). These complex physiological mechanisms likely act independently of oxidative stress and may overshadow the protective role of OBS. Interestingly, ethnicity-based analysis revealed a significant negative association between OBS and gallstones in Mexican Americans, a population at moderate risk of oxidative stress due to a combination of genetic and environmental factors (60). This finding may appear contradictory to the lack of significant associations observed in other high oxidative stress populations, such as older adults and diabetic patients. A plausible explanation lies in genetic predisposition. For instance, the ApoE E4 allele, which is more prevalent in Mexican Americans, confers susceptibility to gallstones. In individuals with this genetic background, the protective effect of OBS may remain within an “effective range,” counteracting moderate oxidative stress levels. Additionally, the high prevalence of sedentary behavior and negative emotional states in Mexican Americans may enhance the manifestation of OBS effects. In contrast, in populations with excessively elevated oxidative stress levels (e.g., diabetic and elderly individuals), the oxidative stress burden may surpass the capacity of OBS to confer protection, reducing its effectiveness in preventing gallstones. Although no non-linear association was observed between OBS and gallstones in our analysis, the findings from subgroup analyses suggest that the interaction of oxidative stress and gallstone risk is highly context-specific. Unlike other chronic diseases, the relationship between OBS and gallstones appears more complex, influenced by genetic, metabolic, and behavioral factors. Future research should aim to investigate the dose–response relationship of OBS under varying oxidative stress conditions, particularly in high-risk subpopulations such as diabetic and elderly patients. Refining and optimizing OBS evaluation systems to account for interindividual variability may help enhance its protective effects and improve preventive strategies for gallstones in diverse populations.

This study has several strengths. First, the analysis utilized a standardized and weighted database, enhancing the reliability and generalizability of the findings. Second, the adoption of a straightforward composite index, such as the OBS, provided an efficient method for assessing oxidative balance at the population level. Third, rigorous control of confounding factors and the inclusion of stratified analyses allowed for a detailed examination of the relationship between OBS and gallstone prevalence across various subgroups. However, several limitations should be noted. First, as a cross-sectional study, it cannot establish a causal relationship between OBS and gallstone prevalence. Second, the dietary data used to calculate OBS were derived from two 24-h dietary recall surveys, which are subject to recall bias and may not reliably reflect long-term dietary patterns. Additionally, the diagnosis of gallstones relied on self-reported data, increasing the risk of underreporting or misclassification. Lastly, the study primarily focused on American participants, which may limit the applicability of the findings to other populations.

## 5 Conclusion

This study identifies a significant inverse association between the OBS and gallstone prevalence, with varying effects across subgroups. These findings highlight the role of oxidative balance in influencing gallstone risk and underscore the complexity of its relationship with population-specific factors.

## Data Availability

Publicly available datasets were analyzed in this study. This data can be found at: https://wwwn.cdc.gov/nchs/nhanes/.
