# Supplementary material for: Higher levels of oxidative balance score linked to lower risk of gallstones: findings from the 2017–2020 National Health and Nutrition Examination Survey
Source: Front Nutr. 2025 Jan 29;12:1521882. doi: 10.3389/fnut.2025.1521882 (PMC11816672; doi:10.3389/fnut.2025.1521882)
Supplement: Supplementary file 1 [file Table_1.docx]

Supplementary Material

# Supplementary Tables

**TABLE S1** Ingredients that make up the oxidative balance score.

| **OBS components** | **Property** | **Female** | | | **Male** | | |
| --- | --- | --- | --- | --- | --- | --- | --- |
|  |  | **0** | **1** | **2** | **0** | **1** | **2** |
| **Dietary OBS** |  |  |  |  |  |  |  |
| Dietary fiber(g/d) | A | ＜9.95 | 9.95-15.50 | ≥15.50 | ＜11.4 | 11.40-17.75 | ≥17.75 |
| Carotene (RE/d) | A | ＜454.83 | 454.83-1602.83 | ≥1602.83 | ＜417 | 417.00-1441.50 | ≥1441.50 |
| Vitamin B6(mg/d) | A | ＜1.15 | 1.15-1.71 | ≥1.71 | ＜1.43 | 1.43-2.16 | ≥2.16 |
| Vitamin B12(mcg/d) | A | ＜2.30 | 2.30-3.94 | ≥3.94 | ＜3.05 | 3.05-5.24 | ≥5.24 |
| Riboflavin (mg/d) | A | ＜1.22 | 1.22-1.74 | ≥1.74 | ＜1.46 | 1.46-2.15 | ≥2.15 |
| Total Folate (mcg/d) | A | ＜224.00 | 224.00-337.16 | ≥337.16 | ＜270.00 | 270.00-409.50 | ≥409.50 |
| Niacin (mg/d) | A | ＜14.92 | 14.92-21.53 | ≥21.53 | ＜18.81 | 18.81-27.97 | ≥27.97 |
| Vitamin C (mg/d) | A | ＜37.60 | 37.60-83.06 | ≥78.50 | ＜28.20 | 38.20-89.65 | ≥89.65 |
| Vitamin E (ATE) (mg/d) | A | ＜5.33 | 5.33-8.21 | ≥8.21 | ＜5.98 | 5.98-9.43 | ≥9.43 |
| Calcium (mg/d) | A | ＜609.00 | 609.00-906.00 | ≥906.00 | ＜720.00 | 720.00-1090.00 | ≥1090.00 |
| Magnesium (mg/d) | A | ＜185.00 | 185.00-261.00 | ≥261.00 | ＜214.00 | 214.00-311.50 | ≥311.50 |
| Zinc (mg/d) | A | ＜6.34. | 6.34.-9.20 | ≥9.20 | ＜7.96 | 7.96-11.87 | ≥11.87 |
| Copper (mg/d) | A | ＜0.70 | 0.70-1.01 | ≥1.01 | ＜0.78 | 0.78-1.19 | ≥1.19 |
| Selenium (mcg/d) | A | ＜68.35 | 68.35-98.23 | ≥98.23 | ＜84.80 | 84.80-126.40 | ≥126.40 |
| Iron (mg/d) | P | ≥12.82 | 8.83-12.82 | ＜8.83 | ≥10.77 | 10.77-15.96 | ＜15.96 |
| Total fat (gm/d) | P | ≥78.38 | 53.83-78.38 | ＜53.83 | ≥97.04 | 64.18-97.04 | ＜64.18 |
|  |  |  |  |  |  |  |  |
| **Lifestyle OBS** |  |  |  |  |  |  |  |
| Physical activity  (MET-minutes/week) | A | ＜225.00 | 225.00-720.00 | ≥720.00 | ＜360.00 | 360.00-1140.00 | ≥1140.00 |
| cotinine(ng/mL) | P | ≥0.09 | 0.01-0.09 | ＜0.01 | ≥0.40 | 0.02-0.40 | ＜0.02 |
| Alcohol(g/d) | P | ≥15.00 | (0, 15.00) | non | ≥30.00 | (0, 30.00) | non |
| Body mass index(kg/m2) | P | ≥30.60 | [23.00, 30.60) | ＜23.00 | ≥29.10 | [22.80, 29.10) | ＜22.80 |

A stood for the antioxidant, P for the pro-oxidant, RE for the retinal equivalent, ATE for the alpha-tocopherol equivalent, and MET for the metabolic equivalent.

**TABLE S2** The definition of the other comorbidities.

Diabetes

1) Medical diagnosis of diabetes as recorded by the patient’s healthcare provider or current use of insulin or diabetic pills.

2) or glycohemoglobin A1c (HbA1c) level higher than 6.5%.

3) or fasting blood glucose level equal to or higher than 126mg/dL.

Hypertension

1) average systolic blood pressure ≥140 mmHg or average diastolic blood pressure ≥90 mmHg by the mean value of at least three times of measurement

2) or self-reported diagnosis of hypertension.

**TABLE S3** Additional baseline characteristics based on the OBS quantile.

| **Characteristic** | **Overall**  **n=5,382** | **Q1**  **n=1264** | **Q2**  **n=1305** | **Q3**  **n=1229** | **Q4**  **n=1584** | ***P*-value** |
| --- | --- | --- | --- | --- | --- | --- |
| Sex |  |  |  |  |  | 0.600 |
| Male | 2,576 (48%) | 583 (46%) | 641 (51%) | 612 (48%) | 740 (49%) |  |
| Female | 2,806 (52%) | 681 (54%) | 664 (49%) | 617 (52%) | 844 (51%) |  |
| Age | 48.58 (17.23) | 48.21 (17.67) | 49.06 (17.22) | 49.73 (17.34) | 47.57 (16.84) | 0.344 |
| Marital |  |  |  |  |  | <0.001 |
| Married/living with partner | 3,174 (63%) | 633 (54%) | 752 (61%) | 777 (67%) | 1,012 (67%) |  |
| Widowed/divorced/separated | 1,213 (18%) | 351 (22%) | 310 (21%) | 253 (16%) | 299 (15%) |  |
| Never married | 995 (19%) | 280 (24%) | 243 (18%) | 199 (17%) | 273 (18%) |  |
| Race |  |  |  |  |  | <0.001 |
| Mexican American | 606 (8.0%) | 91 (6.2%) | 141 (7.5%) | 143 (6.5%) | 231 (11%) |  |
| Non-Hispanic White | 2,063 (65%) | 420 (59%) | 513 (65%) | 513 (69%) | 617 (65%) |  |
| Non-Hispanic Black | 1,394 (10%) | 487 (18%) | 351 (11%) | 257 (8.4%) | 299 (6.9%) |  |
| Other | 1,319 (17%) | 266 (17%) | 300 (16%) | 316 (16%) | 437 (18%) |  |
| PIR | 3.17 (1.64) | 2.71 (1.68) | 3.12 (1.62) | 3.20 (1.62) | 3.47 (1.59) | <0.001 |
| gallstones | 592 (11%) | 157 (14%) | 146 (12%) | 138 (11%) | 151 (9.3%) | 0.049 |
| BMI | 29.81 (7.08) | 30.84 (7.02) | 30.11 (6.95) | 29.59 (7.00) | 29.17 (7.19) | <0.001 |
| Total cholesterol, mg/dL | 185.99 (40.80) | 185.15 (42.83) | 185.19 (39.59) | 185.74 (40.26) | 187.51 (40.53) | 0.630 |
| Total sugars, gm | 101.34 (62.92) | 76.13 (54.56) | 91.33 (59.84) | 106.21 (61.04) | 119.34 (64.78) | <0.001 |
| Total caffe, gm | 169.52± (184.92) | 149.52± (206.66) | 167.56± (189.14) | 187.57± (178.70) | 168.75± (171.44) | <0.001 |
| Total water, gm | 2,928.77± (1,267.93) | 2,148.57± (1,023.13) | 2,715.24± (1,221.13) | 3,015.08± (1,109.53) | 3,469.19± (1,273.79) | <0.001 |
| Sedentary activity, min | 389.71± (630.72) | 370.33± (589.26) | 412.61± (795.94) | 409.05± (731.69) | 370.56± (399.39) | 0.384 |
| Education |  |  |  |  |  | <0.001 |
| Under high school | 830 (9.1%) | 252 (13%) | 203 (9.3%) | 171 (7.8%) | 204 (7.3%) |  |
| High school or equivalent | 1,255 (27%) | 376 (37%) | 330 (29%) | 261 (25%) | 288 (21%) |  |
| Above high school | 3,297 (64%) | 636 (50%) | 772 (62%) | 797 (67%) | 1,092 (72%) |  |
| Smoke |  |  |  |  |  | <0.001 |
| Current smoke | 940 (16%) | 311 (25%) | 257 (17%) | 189 (14%) | 183 (10.0%) |  |
| Former smoke | 1,352 (26%) | 306 (25%) | 319 (26%) | 334 (28%) | 393 (26%) |  |
| Non smoke | 3,090 (58%) | 647 (50%) | 729 (57%) | 706 (58%) | 1,008 (64%) |  |
| Diabetes | 1,134 (16%) | 320 (19%) | 289 (18%) | 248 (14%) | 277 (14%) | 0.021 |
| Hypertension | 2,471 (39%) | 657 (43%) | 605 (41%) | 565 (40%) | 644 (35%) | 0.105 |

All values are presented as mean±SD or as counts (proportion).

OBS: xidative balance score; PIR: poverty income ratio; BMI: body mass index

**TABLE S4** ORs (95% CIs) for gallstones according to the dietary/ lifestyle OBS.

| Characteristic | Model 1 | | | Model 2 | | | Model 3 | | |
| --- | --- | --- | --- | --- | --- | --- | --- | --- | --- |
|  | OR | 95% CI | *P*-value | OR | 95% CI | *P*-value | OR | 95% CI | *P*-value |
| Dietary OBS | | | | | | | | | |
| Continuous | 0.97 | 0.96-0.99 | 0.032 | 0.97 | 0.96-0.99 | 0.017 | 0.97 | 0.95-1.00 | 0.094 |
| OBS quantile |  |  |  |  |  |  |  |  |  |
| Q1 | **—** | **—** |  | **—** | **—** |  | **—** | **—** |  |
| Q2 | 0.83 | 0.59-1.16 | 0.290 | 0.77 | 0.55-1.08 | 0.157 | 0.81 | 0.59-1.11 | 0.212 |
| Q3 | 0.61 | 0.43-0.87 | 0.013 | 0.56 | 0.39-0.81 | 0.007 | 0.58 | 0.38-0.89 | 0.019 |
| Q4 | 0.73 | 0.53-1.01 | 0.077 | 0.73 | 0.54-0.98 | 0.050 | 0.77 | 0.53-1.12 | 0.196 |
| P for trend |  |  | 0.045 |  |  | 0.036 |  |  | 0.150 |
| Lifestyle OBS | | | | | | | | | |
| Continuous | 0.86 | 0.78-0.95 | 0.009 | 0.88 | 0.78-0.98 | 0.038 | 0.88 | 0.79-0.99 | 0.042 |
| OBS quantile |  |  |  |  |  |  |  |  |  |
| Q1 | **—** | **—** |  | **—** | **—** |  | **—** | **—** |  |
| Q2 | 0.80 | 0.51-1.27 | 0.369 | 0.76 | 0.49-1.18 | 0.242 | 0.74 | 0.50-1.11 | 0.164 |
| Q3 | 0.70 | 0.46-1.06 | 0.109 | 0.60 | 0.39-0.93 | 0.033 | 0.58 | 0.38-0.90 | 0.023 |
| Q4 | 0.54 | 0.33-0.88 | 0.021 | 0.57 | 0.35-0.94 | 0.038 | 0.60 | 0.37-0.96 | 0.046 |
| P for trend |  |  | 0.010 |  |  | 0.031 |  |  | 0.047 |

OR: odds ratio, CI: confidence interval

Model 1: Unadjusted

Model 2: Adjusted for age, and race,

Model 3: Adjusted for age, race, marital, poverty income ratio, education, total sugars, total caffe, total water, diabetes, hypertension.

**TABLE S5** Sensitivity analyses of gallstones outcomes by sequential elimination of individual OBS components.

| **OBS component excluded** | **Gallstones** | | |
| --- | --- | --- | --- |
|  | **OR (95% CI)** | **p-value** | **P for trend** |
| Dietary fiber |  |  |  |
| Continuous | 0.97(0.94-0.99) | 0.030 |  |
| Q1 | 1(Reference) |  |  |
| Q2 | 0.88(0.64-1.22) | 0.477 |  |
| Q3 | 0.71(0.52-0.96) | 0.038 |  |
| Q4 | 0.58(0.41-0.83) | 0.006 | 0.013 |
| Carotene |  |  |  |
| Continuous | 0.97(0.95-0.99) | 0.039 |  |
| Q1 | 1(Reference) |  |  |
| Q2 | 0.74(0.57-0.95) | 0.028 |  |
| Q3 | 0.67(0.50-0.91) | 0.018 |  |
| Q4 | 0.60(0.41-0.87) | 0.014 | 0.033 |
| Vitamin B6 |  |  |  |
| Continuous | 0.97(0.95-0.99) | 0.040 |  |
| Q1 | 1(Reference) |  |  |
| Q2 | 0.87(0.63-1.20) | 0.413 |  |
| Q3 | 0.67(0.47-0.95) | 0.034 |  |
| Q4 | 0.56(0.39-0.80) | 0.004 | 0.010 |
| Vitamin B12 |  |  |  |
| Continuous | 0.97(0.95-0.99) | 0.034 |  |
| Q1 | 1(Reference) |  |  |
| Q2 | 0.88(0.67-1.14) | 0.348 |  |
| Q3 | 0.60(0.43-0.85) | 0.007 |  |
| Q4 | 0.68(0.46-1.02) | 0.076 | 0.046 |
| Riboflavin |  |  |  |
| Continuous | 0.96(0.95-0.99) | 0.028 |  |
| Q1 | 1(Reference) |  |  |
| Q2 | 0.88(0.66-1.18) | 0.416 |  |
| Q3 | 0.68(0.50-0.94) | 0.028 |  |
| Q4 | 0.57(0.41-0.80) | 0.003 | 0.007 |
| Total Folate |  |  |  |
| Continuous | 0.97(0.95-0.99) | 0.034 |  |
| Q1 | 1(Reference) |  |  |
| Q2 | 0.74 (0.52-1.05) | 0.112 |  |
| Q3 | 0.66(0.47-0.93) | <0.028 |  |
| Q4 | 0.55(0.40-0.75) | <0.001 | 0.008 |
| Niacin |  |  |  |
| Continuous | 0.97(0.95-0.99) | 0.048 |  |
| Q1 | 1(Reference) |  |  |
| Q2 | 0.71(0.49-1.03) | 0.084 |  |
| Q3 | 0.33(0.20-0.54) | <0.001 |  |
| Q4 | 0.15(0.08-0.29) | <0.001 | <0.001 |
| Vitamin C |  |  |  |
| Continuous | 0.97(0.95-0.99) | 0.036 |  |
| Q1 | 1(Reference) |  |  |
| Q2 | 0.80(0.60-1.05) | 0.131 |  |
| Q3 | 0.66(0.46-0.93) | 0.026 |  |
| Q4 | 0.60(0.40-0.91) | 0.025 | 0.037 |
| Vitamin E (ATE) |  |  |  |
| Continuous | 0.97(0.95-0.99) | 0.041 |  |
| Q1 | 1(Reference) |  |  |
| Q2 | 0.77(0.56-1.04) | 0.100 |  |
| Q3 | 0.73(0.51-1.04) | 0.099 |  |
| Q4 | 0.60(0.42-0.85) | 0.009 | 0.032 |
| Calcium |  |  |  |
| Continuous | 0.97(0.94-0.99) | 0.031 |  |
| Q1 | 1(Reference) |  |  |
| Q2 | 0.72(0.54-0.95) | 0.030 |  |
| Q3 | 0.69(0.49-0.97) | 0.043 |  |
| Q4 | 0.60(0.42-0.86) | 0.010 | 0.033 |
| Magnesium |  |  |  |
| Continuous | 0.97(0.94-0.99) | 0.031 |  |
| Q1 | 1(Reference) |  |  |
| Q2 | 0.86(0.61-1.22) | 0.426 |  |
| Q3 | 0.69(0.49-0.97) | 0.044 |  |
| Q4 | 0.62(0.42-0.90) | 0.019 | 0.037 |
| Zinc |  |  |  |
| Continuous | 0.97(0.94-0.99) | 0.040 |  |
| Q1 | 1(Reference) |  |  |
| Q2 | 0.82(0.62-1.09) | 0.199 |  |
| Q3 | 0.67 (0.46-0.97) | 0.049 |  |
| Q4 | 0.59(0.40-0.85) | 0.010 | 0.021 |
| Copper |  |  |  |
| Continuous | 0.98(0.96-1.01) | 0.479 |  |
| Q1 | 1(Reference) |  |  |
| Q2 | 0.93(0.67-1.28) | 0.666 |  |
| Q3 | 0.67(0.48-0.93) | 0.026 |  |
| Q4 | 0.62(0.43-0.90) | 0.019 | 0.023 |
| Selenium |  |  |  |
| Continuous | 0.97(0.94-0.99) | 0.042 |  |
| Q1 | 1(Reference) |  |  |
| Q2 | 0.70(0.51-0.96) | 0.039 |  |
| Q3 | 0.66(0.48-0.90) | 0.016 |  |
| Q4 | 0.58(0.40-0.84) | 0.007 | 0.032 |
| Iron |  |  |  |
| Continuous | 0.97(0.95-0.99) | 0.049 |  |
| Q1 | 1(Reference) |  |  |
| Q2 | 0.76(0.57-1.02) | 0.085 |  |
| Q3 | 0.73(0.53-1.00) | 0.068 |  |
| Q4 | 0.61(0.42-0.89) | 0.018 | 0.038 |
| Total fat |  |  |  |
| Continuous | 0.97(0.95-0.99) | 0.037 |  |
| Q1 | 1(Reference) |  |  |
| Q2 | 0.85(0.64-1.14) | 0.313 |  |
| Q3 | 0.70(0.49-0.99) | 0.061 |  |
| Q4 | 0.60(0.42-0.85) | 0.008 | 0.021 |
| Physical activity |  |  |  |
| Continuous | 0.97(0.95-0.99) | 0.039 |  |
| Q1 | 1(Reference) |  |  |
| Q2 | 0.91(0.68-1.22) | 0.555 |  |
| Q3 | 0.61(0.42 -0.88) | 0.015 |  |
| Q4 | 0.65(0.43-0.96) | 0.044 | 0.036 |
| Cotinine |  |  |  |
| Continuous | 0.97(0.95-0.99) | 0.045 |  |
| Q1 | 1(Reference) |  |  |
| Q2 | 0.80(0.63-1.02) | 0.088 |  |
| Q3 | 0.60(0.46-0.79) | 0.001 |  |
| Q4 | 0.60(0.42-0.86) | 0.010 | 0.018 |
| Alcohol |  |  |  |
| Continuous | 0.97(0.95-0.99) | 0.022 |  |
| Q1 | 1(Reference) |  |  |
| Q2 | 0.84(0.68-1.05) | 0.153 |  |
| Q3 | 0.62(0.42-0.91) | 0.023 |  |
| Q4 | 0.55(0.39-0.78) | 0.002 | 0.009 |
| Body mass index |  |  |  |
| Continuous | 0.97(0.95-0.99) | 0.060 |  |
| Q1 |  |  |  |
| Q2 | 0.78(0.56-1.10) | 0.182 |  |
| Q3 | 0.68(0.48-0.96) | 0.042 |  |
| Q4 | 0.67(0.47-0.96) | 0.041 | 0.060 |

CI = Confidence Interval, OR = Odds Ratio

Model has been adjusted for age, race, marital, poverty income ratio, education, total sugars, total caffe, total water, diabetes, hypertension.

# Supplementary Figures


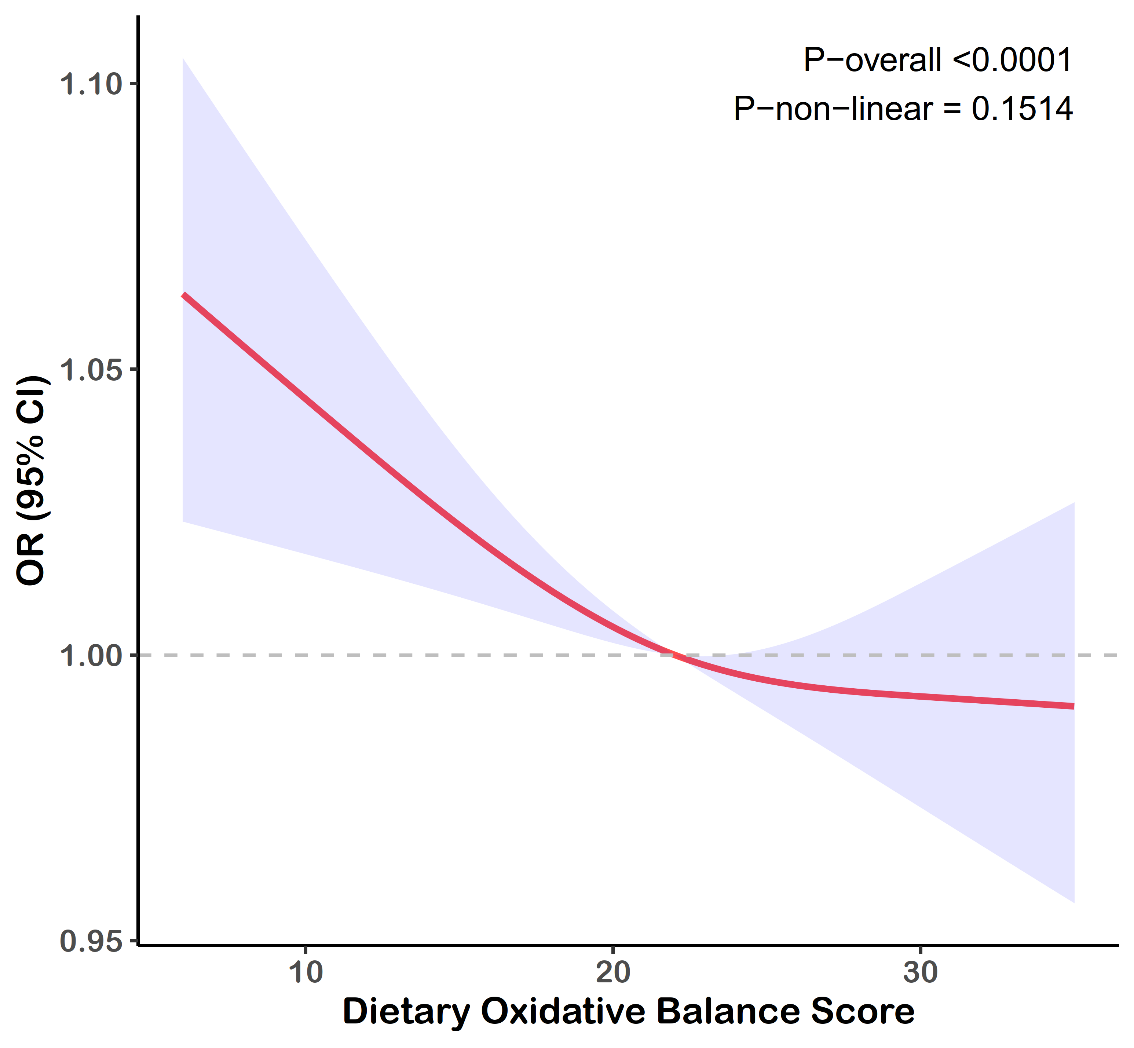


**FIGURE S1** RCS curve for the association between the Dietary OBS and the risk of gallstones. Lines represent odds ratios, and areas represent 95% confidence intervals. The model was adjusted for age, race, marital, poverty income ratio, education, total sugars, total caffe, total water, diabetes, and hypertension.


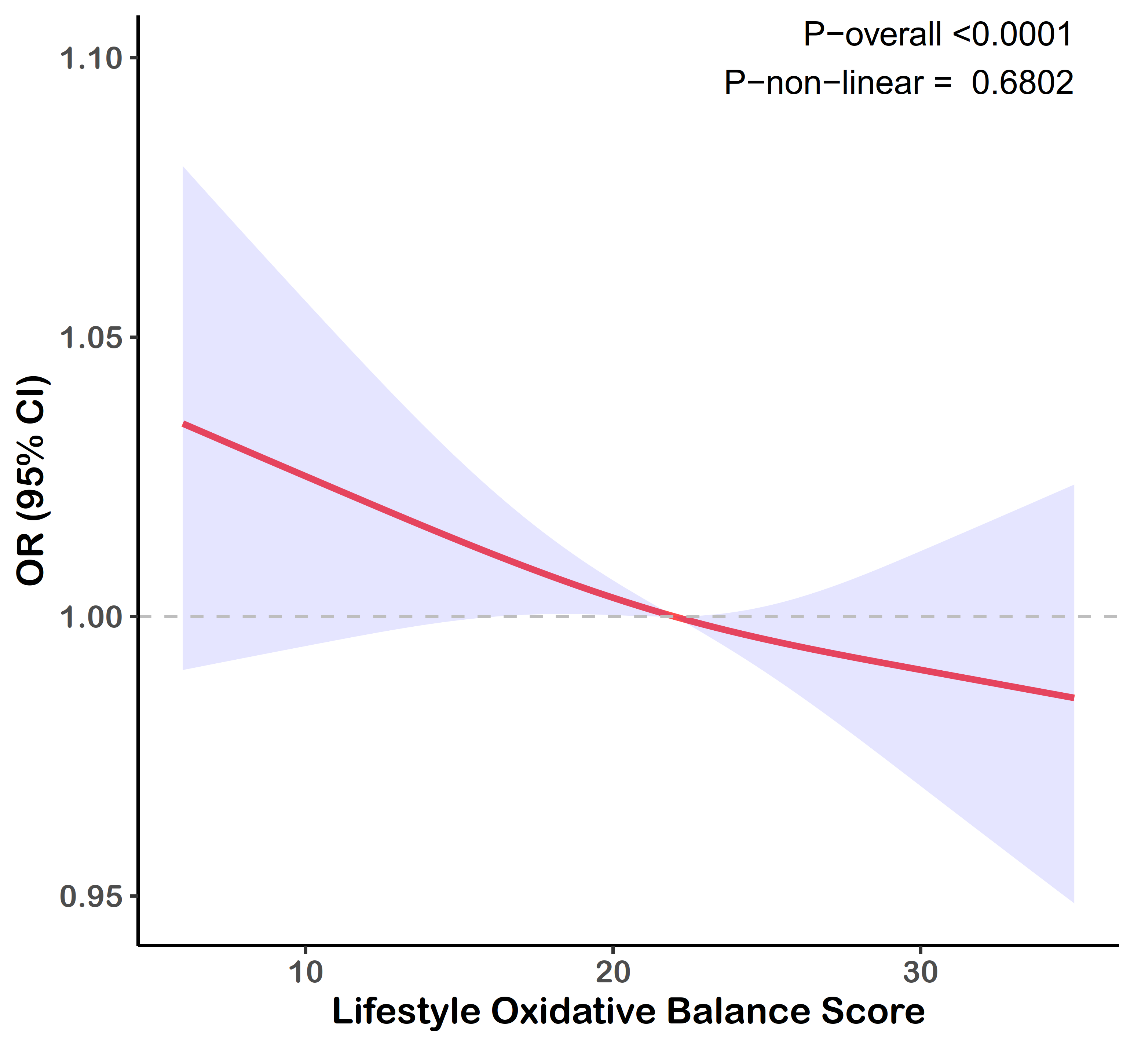


**FIGURE S2** RCS curve for the association between the Lifestyle OBS and the risk of gallstones. Lines represent odds ratios, and areas represent 95% confidence intervals. The model was adjusted for age, race, marital, poverty income ratio, education, total sugars, total caffe, total water, diabetes, and hypertension.
